# Improving the performance of social health insurance system through increasing outpatient expenditure reimbursement ratio: a quasi-experimental evaluation study from rural China

**DOI:** 10.1186/s12939-018-0799-8

**Published:** 2018-06-25

**Authors:** Yudong Miao, Jianqin Gu, Liang Zhang, Ruibo He, Sandeep Sandeep, Jian Wu

**Affiliations:** 1 Henan Provincial People’s Hospital, 7 Weiwu Road, Zhengzhou, 450003 Henan Province China; 2 School of Health Policy and Management, Nanjing Medical University, Nanjing, China; 3 School of Medicine and Health Management, Tongji Medical College, Huazhong University of Science & Technology, Wuhan, China; 4 College of Public Health, Zhengzhou University, Zhengzhou, China

**Keywords:** Inpatient, Outpatient, Social health insurance, Performance, Hypertension

## Abstract

**Background:**

China has set up a universal coverage social health insurance system since the 2009 healthcare reform. Due to the inadequate funds, the social health insurance system reimbursed the inpatient expenditures with much higher ratio than outpatient expenditure. The gap in reimbursement ratios resulted in a rapid rising hospitalization rate but poor health outcomes among the Chinese population. A redistribution of social health insurance funds has become one of the main challenges for the performance of Social Health Insurance.

**Methods:**

Two comparable counties, Dangyang County and Zhijiang County, in Hubei Province of China, were sampled as the intervention group and the control group, respectively. The Social Health Insurance Management Department of the intervention group budgeted 600 yuan per capita per year to the patients with 3rd stage hypertension to cover their outpatient expenditures. The outpatient spending in the control group were paid out-of-pocket. The inpatient expenditures reimbursement policies in both groups were not changed. Besides, the Social Health Insurance Management Department of the intervention group budgeted 100 yuan per patient per year to township physicians and hospitals to provide health management services for the patients. While, the health management services in the control group were still provided by health workers. A Propensity Score Matching model and Difference-in-differences model were used to estimate the net effects of the intervention in dimensions of medical services utilization, medical expenditures, SHI reimbursement, and health outcomes.

**Results:**

One thousand, six hundred and seventy three pairs of patients were taken as valid subjects to conduct Difference-in-differences estimation after the Propensity Score Matching. The net intervention effect is to increase outpatient frequency by 3.3 (81.0%) times (*P* < 0.05), to decrease hospitalization frequency by 0.075 (− 60.0%) times (*P* < 0.05), and to increase the per capita total medical service utilization frequency by 3.225 (76.8%) times (*P* < 0.05). The per capita total medical expenditure decreased 394.2 (− 27.7%) yuan. The SHI reimbursed 90.3 yuan more per capita for the outpatient spending, but the per capita inpatient expenditure reimbursement and per capita total medical expenditure reimbursement decreased significantly by 282.6 (− 44.0%) yuan and 192.3 (− 28.5%) yuan, respectively (*P* < 0.05). The intervention reduced the per capita inpatient out-of-pocket expenditure and the per capita total out-of-pocket expenditure by 192.8 (− 36.7%) yuan and 201.9 (− 29.9%) yuan, respectively (*P* < 0.05). The intervention significantly decreased the diastolic blood pressure of the intervention group by 2.9 mmHg (*P* < 0.05) but had no significant impact on the systolic blood pressure (− 7.9 mmHg, *P* > 0.05).

**Conclusion:**

For China and countries attempting to establish a universal coverage SHI with inadequate funds, inpatient services were expensive but might not produce good health outcomes. Outpatient care for patients with chronic diseases should be fundamental, and outpatient expenditures should be reimbursed with a higher ratio.

## Background

As a country with 1.4 billion people, China’s healthcare reform affects global health, not only because the large population comprise a fifth of the world population, but also because its innovations and experiences will be helpful and influential for other countries [1]. Thanks to continued economic growth, China has invested more and more money from as early as 20 years ago to build its universal coverage health system [2–4]. Since the central government decided to promote the health system reform in 1997, China’s health service system has made remarkable progresses in trying to set up family doctor system [5], increasing the proportion of total health expenditure in gross domestic product [6], configuring diagnosis and treatment equipment for medical institutions [7], promoting the construction of medical information platform throughout the country [8, 9], and strengthening the supervision of health services delivery [10]. Meanwhile, health policymakers realized that the escalating medical costs and lack of insurance coverage often cause transient poverty for many Chinese families [11]. Therefore, China has gradually established a social health insurance system (SHI) consisting of the Basic Medical Insurance System for Urban Workers (since 1998), the New Rural Cooperative Medical Service System for Rural Residents (since 2003), and the Basic Medical Insurance System for Urban Residents (since 2007) [12]. This impressive system has covered more than 95% of Chinese residents and reimbursed approximately half of their medical expenditures nowadays [13–15].

According to the initial goal of China’s healthcare reform, all the residents would be able to equitably acquire quality health services with little out-of-pocket expenses [16]. Considering the outpatient care is significantly less costly than inpatient treatment [17–19], the inpatient medical expenditures reimbursement ratio of the insured patients (> 50%) is set to be much higher than outpatient medical expenditures (zero or very low). The dramatic gap between inpatient expenditure reimbursement ratio (higher) and outpatient expenditure reimbursement ratio (lower) obviously affected the medical choice of the diseased population [20].

**Fig. 1:**
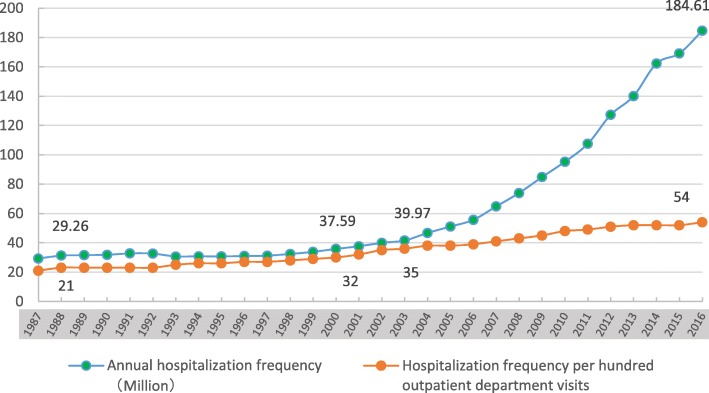
The trends of hospitalization services utilization in China over the past 30 years: 1987 to 2016

In general, most of the health needs could be met through outpatient care in many western countries [21]. However, in the context of Chinese SHI policy design, more and more patients require hospitalization from their attending doctors rather than their actual needs of medical services [22]. Once their admission requests are rejected, many patients who have slight disease symptoms but actually need outpatient care would try their best to avoid seeking any outpatient services (usually not covered by SHI) to save the out-of-pocket expenses. Their underuse of outpatient services often in return leads to more serious conditions for which hospitalization treatment is unavoidable [23]. The National Health and Family Planning Commission of China reported that during the past 30 years, the annual number of hospitalization increased from 29.26 million to 184.61 million (per year growth rate was 17.7%); the inflection point occurred around the year 2003, from when China began to accomplish the establishment of the universal coverage SHI system. During the 15 years of 1987 to 2001, the annual number of hospitalization increased from 29.26 million to 37.59 million (per year growth rate was 1.9%); however, during the next 15 years of 2002 to 2016, the annual number of hospitalization increased from 39.97 million to 184.61 million (per year growth rate was 24.1%). Meanwhile, the sustained growth of hospitalization frequency per hundred outpatient department visits revealed that hospitalization growth rate has been faster than outpatient care. Figure 1 illustrated the evolution of China’s hospitalization services utilization in the past 30 years [24].

**Fig. 2:**
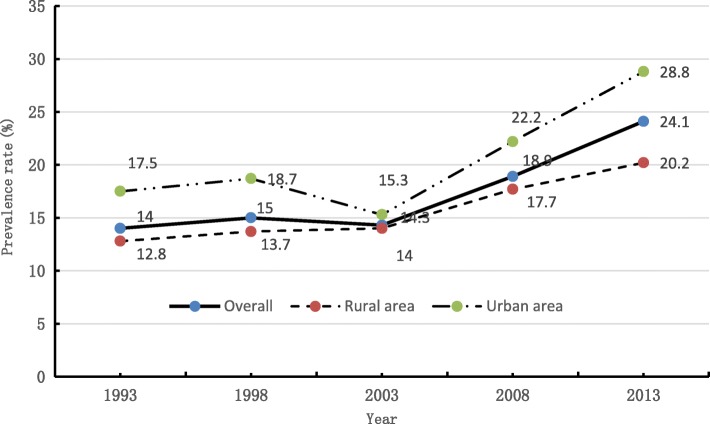
The two-week prevalence rate among Chinese from 1993 to 2013

The SHI induced “seeking inpatient services or seeking no health services” among the population, ironically but expected, leads to the rapid rising hospitalization rate but a significant increasing trend in the two-week prevalence rate (poor health outcomes) [25]. Figure 2 illustrated that the inflection also occurred around the year 2002 to 2003.

Although it is still debatable to what extent China’s rapid rising hospitalization is reasonable, inpatient services cost more than 90% of SHI funds in the context of SHI funds have been increasing 37.9% per year in recent 20 years. The significant cost of hospitalization makes it impossible for SHI to improve outpatient expenditures reimbursement ratio. The poor health outcomes among the Chinese exposed the adverse effects of the SHI reimbursement policy defects, and redistribution of Chinese SHI is necessary for better SHI performance. Similar SHI policy design defects may occur in countries attempting to establish a universal coverage SHI system with inadequate funds like China. In this study, we will present an SHI performance-oriented reform in Dangyang County of Hubei Province, China. The project aimed to reduce the hospitalization rate among patients with grade 3 hypertension, as well as to improve their health outcomes, through increasing outpatient service reimbursement ratio. The project process will be introduced in detail, and the intervention impacts on medical service utilization, medical expenditure and reimbursement, and patient health outcomes will be estimated. The findings of this study not only provide a solution for China’s rapid rising hospitalization rate and poor SHI performance, but also help to enlighten global SHI policymakers to avoid the dilemma China is now experiencing.

## Methods

### Study time and settings

**Fig. 3:**
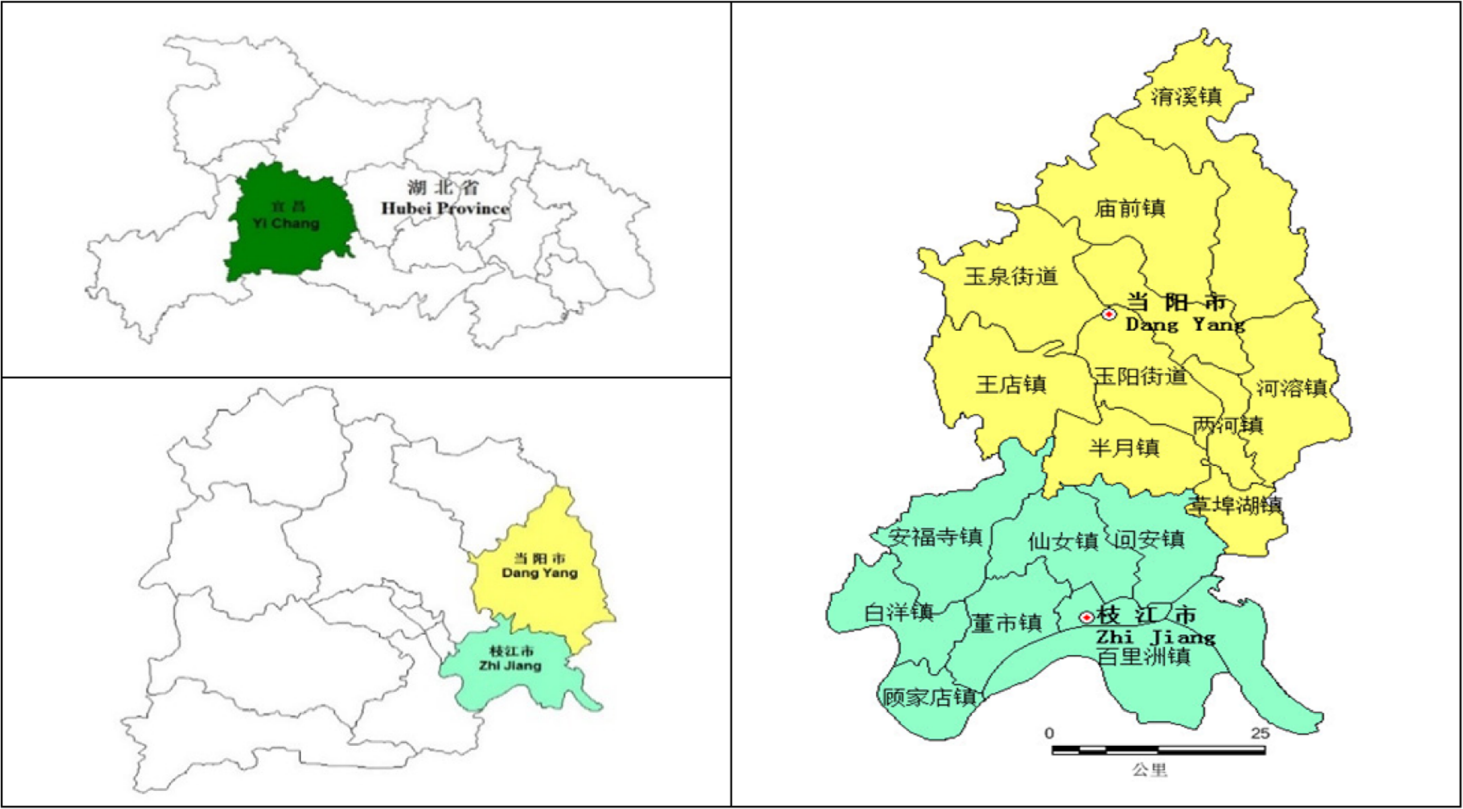
Study settings: location of Dangyang County and Zhijiang County

The whole study was started in July 2015 and accomplished in March 2017. The intervention measures were implemented during January 2016 to December 2016. A quasi-experimental evaluation method was used to estimate the SHI performance-oriented reform on medical service utilization, medical expenditure, and patient health outcome. Two similar and neighboring counties (shown in Fig. 3), Dangyang County and Zhijiang County, in Hubei Province of China, were sampled as the intervention group and the control group, respectively. Both counties were comparable in administrative districts, population, aging population rate, urbanization rate and per capita annual income (see Table 1).

**Table 1:** Indicators of Dangyang and Zhiijang County

Indicators	Dangyang	Zhijiang
Location	Contiguous to Zhijiang	Contiguous to Dangyang
Administrative districts	10 towns	9 towns
Population(thousand)	486	496
Aging population rate (%)	18.7	15.2
Urbanization rate (%)	50.0	54.0
Per capita annual income (urban/rural, yuan)	27,433/16928	23,675/15,285

### Participant recruitment

#### Patient sampling

**Fig. 4:**
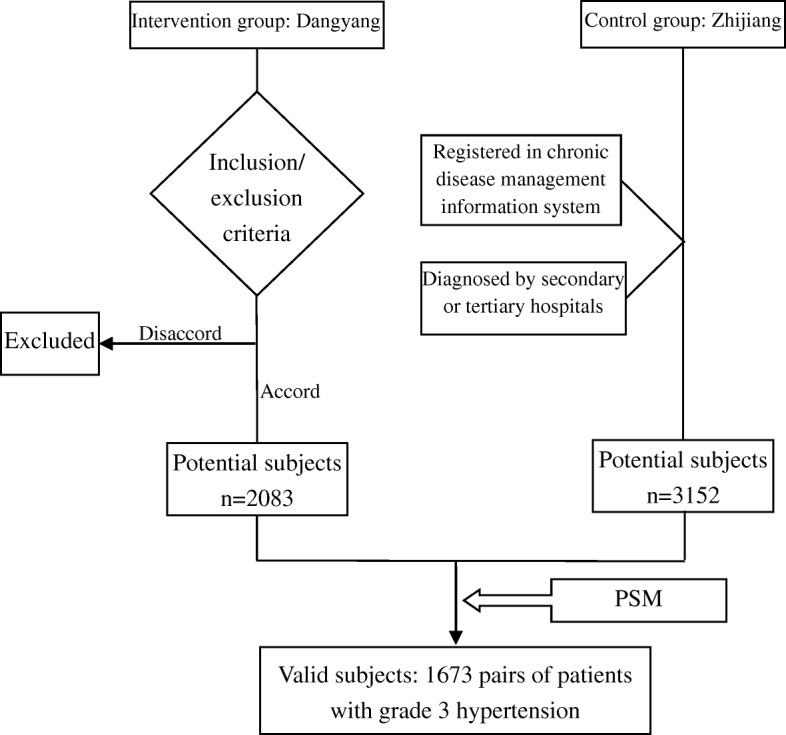
Patient sampling flow chart

A two-stage sampling method was used to obtain comparable valid subjects from the intervention group and control group. At the first stage, a cluster sampling method was used to select observation subjects by the inclusion and exclusion criteria of this study. The potential participants at the first stage were patients who were (1) covered by SHI; (2) local census register population and registered in chronic disease management system of local township hospital; (3) willing to participate and sign the informed consent; (4) diagnosed by secondary or tertiary hospitals with grade 3 hypertension, or hypertension complicated with left ventricular hypertrophy, cerebral hemorrhage, cerebral thrombosis, cerebral lacunar infarction, coronary atherosclerosis and microvascular disease, renal failure, fundus retinal arteriole, exudation and bleeding and other symptoms. Patients who (1) were not permanent residents of the sampled counties; (2) refused to be followed up by medical staff participating this project; (3) had a life expectancy of fewer than 1 years, or died during the project, were excluded from the study. The first-stage cluster sampling enrolled 2083 and 3152 patients with grade 3 hypertension into the intervention and control group, respectively. To control the confounders, medical service utilization and medical expenditures influencing factors, including sex, age, incomes and health conditions, were collected. A PSM model was then used to conduct a comparability match between both groups. The model produced 1673 patients from the intervention group and the matched 1673 patients from the control group. The 1673 pairs of patients were taken as valid subjects for statistical analysis. The first stage sampling was conducted in December 2015. After the intervention, we conducted a follow-up survey to collect end point data. The flow chart of patient sampling was shown in Fig. 4.

### Physician contract

Each township hospitals in Dangyang county was urged by the Social Health Insurance Management Department of Dangyang Health Bureau to recommend at least 2 township hospital physicians to participate in the project. Physicians signed informed consent would be contracted by Medical Insurance Management Department. The contracted physicians provided services according to the requirement of the project. The SHI fund of the intervention group budgeted 100 yuan per patient per year to the township hospitals and the contracted doctors as project recompense. The performance of the contracted physicians would be evaluated by Social Health Insurance Management Department of Dangyang at the end of the year. The performance evaluation results decided the actual compensated fund for each contracted physician. To motivate the contracted physicians, the Social Health Insurance Management Department of Dangyang required township hospitals to give at least 50% of the actual recompense from the SHI fund to these physicians.

## Intervention

### SHI increasing outpatient expenditure reimbursement ratio

In the implementation of this project, the outpatient expenditure reimbursement ratio was increased substantially. The SHI fund of the intervention group budgeted 600 yuan per patient per year to the enrolled patients to cover their outpatient services for hypertension or hypertension complications. When seeking outpatient services for hypertension or hypertension complications, the patients paid for the medical expenditures out-of-pocket. The out-of-pocket expenses will be reimbursed next month, and the money will be returned to debit cards of the patients. If the total reimbursement of a patient exceeded 600 yuan within the year, the rest outpatient services expenditures would be paid out-of-pocket. Correspondingly, the budget would be retracted by SHI fund if it was not finished at the end of the year. Outpatient expense of patients with grade 3 hypertension was not covered by SHI in Zhijiang County.

No adjustment was made in the inpatient expenditure reimbursement policy of both groups. The average inpatient reimbursement ratio was about 50%.

### Health management services provided by contracted township hospital physicians

This SHI performance-oriented interventional study aimed to obtain multifaceted positive outcomes in utilization of medical services, the efficiency of SHI fund and health outcomes of patients. The contracted physicians in the intervention group followed up the enrolled patients each quarter in ways of household follow-up or outpatient face-to-face follow-up. The services provided in the follow-up included blood pressure monitoring, lifestyle guidance, complication symptoms assessment, drug prescriptions evaluation and adjustment, and seeking medical treatment suggestions. Also, the contracted physicians informed the patients the outpatient expenditures reimbursement policy in the implementation of the project. After each follow-up, the physician would fill out a follow-up table and get the signature of the patient. The physician would also take a group photo together with the patient as proof of follow-up service provision. The completed tables and group photos were taken as the important basis for the performance assessment of the contracted physicians. There was no contracted physician providing health management services for patients with grade 3 hypertension.

## Project oversight

The performance of the contracted physicians was evaluated according to Performance Assessment Rules for Chronic Disease Outpatient Management of Dangyang County. Physicians scored > = 90 points (0–100) would be recompensed 100 yuan per patient per year. The score was reduced by 1 point, and the recompense would be deducted by 1%.

## Data collection

Baseline information of the enrolled patients was collected during November 10 to December 20, 2015. Sex, age, and blood pressure were obtained from their latest medical record in township hospitals or county hospitals. Income was collected through a telephone survey. Data related to medical service utilization frequency, medical expenditure and reimbursement was extracted from SHI Management Information System. Follow-up survey was conducted in the same way during January 10 to February 6, 2015, but income was not collected.

## Statistical analysis

### Propensity score matching (PSM) model [26–29]

A greedy matching procedure with calipers set at 0.1 standard deviations of the probit of the propensity score was used to create matched pairs (model setting was shown in Appendix). For each propensity score, we computed the standardized differences for sex, age, annual income, diastolic blood pressure (DBP) and systolic blood pressure (SBP) in the matched sample. For dichotomous and continuous variables, the definitions of standardized difference was also seen in Appendix.

### Difference-in-differences (DID) model [30–32]

Basing on the matched subjects through PSM, this study used the fixed effect estimation method of DID to estimate the differences of medical services utilization, medical expenditure, SHI reimbursement expenditure, and patient blood pressures between the intervention group and control group to assess the net effects of the intervention. DID analysis is a quasi-experimental method which had been widely used to evaluate project outcomes, mostly in public health projects. The method pretends to capture the effects related to some treatment or event through time, between a treatment group and a control group. For the observed individual *I*, the basic settings for DID model were:

$$ {y}_i={\beta}_0+{\beta}_1\cdotp {time}_i+\gamma \cdotp {group}_i+\delta \cdotp {group}_i\cdotp {time}_i+{\varepsilon}_{i.} $$

The estimated coefficients in this linear regression had the following interpretation:

- $\hat{\upbeta_0}$: Mean outcome for the control group at baseline.
- $\hat{\upbeta_0}$*+*$\hat{\upbeta_1}$: Mean outcome for the control group at follow-up.
- $\hat{\upgamma}$: The single difference between treatment and control groups at baseline.
- $\hat{\upbeta_0}$*+*$\hat{\upgamma}$: Mean outcome for the treatment group at baseline.
- $\hat{\upbeta_0}$*+*$\hat{\upbeta_1}$*+*$\hat{\upgamma}$*+*$\hat{\updelta}$: Mean outcome for the treatment group at follow-up.
- $\hat{\updelta}$: The DID or impact of the intervention.
- *ε*_*i*_:The random error.

A value of *P* < 0.05 was considered as statistically significant. All data analysis was performed using STATA statistical software version 11.0 (http://www.stata.com/stata11/).

## Ethical issues

All enrolled patients with grade 3 hypertension in the control group were given a written informed consent. Information related to their sex, age, health status was anonymously extracted from the SHI Management Information System or their latest medical records.

## Results

### Valid observation subjects through PSM

Table 2 showed that the potential subjects of both groups were statistically different in sex, age and DBP (*P* < 0.05). The PSM produced 1673 pairs of the subject with comparable sex, age, annual income, DBP and SBP (*P* > 0.05). All standardized differences for the covariate in the matched sample were <10%, suggesting balance in the given variables between the intervention and control groups.

**Table 2:** Valid observation subjects through PSM and balance between subjects in matched samples

Variables	Before PSM		Standardized difference (%)	*P*	Variables	After PSM		Standardized difference (%)	*P*
	Intervention group (*n* = 2083)	Control group (*n* = 3152)				Intervention group (*n* = 1673)	Control group (*n* = 1673)		
Sex (male, %)	49.1	51.9	−5.6	0.048	Sex (male, %)	52.4	51.7	1.4	0.678
Age (year)	66.4 ± 7.9	62.9 ± 11.4	35.7	0.021	Age (year)	65.0 ± 5.1	64.5 ± 7.3	7.9	0.307
Annual income (yuan)	12,901 ± 20,705	13,865 ± 19,244	−4.8	0.143	Annual income (yuan)	12,805 ± 14,429	12,863 ± 12,050	−0.4	0.992
Diastolic pressure (mmHg)	115.6 ± 33.2	111.9 ± 39.0	10.2	0.039	Diastolic pressure (mmHg)	114.9 ± 29.7	112.6 ± 34.6	7.1	0.420
Systolic pressure (mmHg)	198.0 ± 65.7	201.1 ± 68.2	−4.6	0.122	Systolic pressure (mmHg)	197.5 ± 39.5	197.2 ± 30.1	0.9	0.855

### Frequency of per capita annual medical service utilization

Table 3 showed the effects of the intervention on outpatient, inpatient, and total medical service utilization. At baseline, there was no statistical difference in frequencies of outpatient and overall visits between both groups (4.180 vs. 4.072). The average hospitalization frequency of the intervention group is 0.125 times, which was significantly higher than the 0.063 times of the control group. At follow-up, the outpatient frequency of the intervention group increased from 4.072 times to 7.353 times and was significantly higher than the 4.161 times of the control group. The DID result showed that the policy intervention effect was to increase outpatient frequency by 3.3 (81.0%) times (*P* < 0.05), and to decrease hospitalization frequency by 0.075 (− 60.0%) times (*P* < 0.05). The per capita total medical service utilization frequency of the intervention group was 7.427 times, which was significantly higher than the 4.248 times of the control group (*P* < 0.05). The DID result showed that the policy intervention effect was to increase the per capita total medical service utilization frequency by 3.225 (76.8%) times (*P* < 0.05).

**Table 3:** DID estimation on frequencies of medical services utilization

Outcome variable (y)	Baseline (1673 vs. 1673)			Follow-up (1673 vs. 1673)			
	Control $\left({\widehat{\upbeta}}_0\right)$	Intervention $\left({\widehat{\upbeta}}_0+\widehat{\gamma}\right)$	Diff (BL) $\left(\widehat{\gamma}\right)$	Control $\left({\widehat{\upbeta}}_0+{\widehat{\upbeta}}_1\right)$	Intervention $\left({\widehat{\upbeta}}_0+{\widehat{\upbeta}}_1+\widehat{\gamma}+\widehat{\updelta}\right)$	Diff (FU) $\left({\widehat{\gamma}}_0+\widehat{\updelta}\right)$	DIFF-IN-DIFF $\left(\widehat{\updelta}\right)$
Outpatient	4.180	4.072	−0.108	4.161	7.353	3.192	3.3
*P*			0.115			0.041	0.008
Inpatient	0.063	0.125	0.062	0.087	0.074	−0.013	−0.075
*P*			0.000			0.095	0.000
Total medical services	4.243	4.197	−0.046	4.248	7.427	3.179	3.225
*P*			0.862			0.003	0.001

### Per capita annual medical expenditure

The per capita outpatient expenditure, inpatient expenditure and total medical expenditure of both groups were compared to evaluate the net policy effects (see Table 4). At baseline, the per capita outpatient expenditures of both groups were comparable (*P* > 0.05). The per capita inpatient expenditure of the intervention group (1167.6 yuan) was significantly higher than the control group (818.1 yuan, *P* < 0.05). The per capita total medical expenditure of the intervention group was 1423 yuan, which was higher than the 1087.3 yuan of the control group (*P* < 0.05). At follow-up, the per capita outpatient expenditures of both groups were also comparable (*P* > 0.05). However, the per capita inpatient expenditure and total medical expenditure of the control group were significantly higher than the intervention group (910.9 vs. 785.0, 1174.7 vs. 1116.2, *P* < 0.05). The DID estimation indicated that the intervention increased per capita outpatient expenditure by 81.2 (31.8%) yuan but decreased the per capita inpatient expenditure by 475.4 (− 40.7%) yuan. In total, the project reduced the per capita total medical expenditure by 394.2 (− 27.7%) yuan.

**Table 4:** DID estimation on per capita annual medical expenditure

Outcome variable (y)	Baseline (1673 vs. 1673)			Follow-up (1673 vs. 1673)			
	Control $\left({\widehat{\upbeta}}_0\right)$	Intervention $\left({\widehat{\upbeta}}_0+\widehat{\gamma}\right)$	Diff (BL) $\left(\widehat{\gamma}\right)$	Control $\left({\widehat{\upbeta}}_0+{\widehat{\upbeta}}_1\right)$	Intervention $\left({\widehat{\upbeta}}_0+{\widehat{\upbeta}}_1+\widehat{\gamma}+\widehat{\updelta}\right)$	Diff (FU) $\left({\widehat{\gamma}}_0+\widehat{\updelta}\right)$	DIFF-IN-DIFF $\left(\widehat{\updelta}\right)$
Outpatient	269.2	255.4	−13.8	263.8	331.2	67.4	81.2
*P*			0.452			0.095	0.048
Inpatient	818.1	1167.6	349.5	910.9	785.0	−125.9	−475.4
*P*			0.009			0.003	0.027
Total	1087.3	1423.0	335.7	1174.7	1116.2	−58.5	−394.2
*P*			0.015			0.043	0.013

### SHI per capita annual reimbursement expenditure

As the trigger of the reform, the SHI attempted to explore a more efficient way to use medical insurance funds. This study analyzed the per capita outpatient expense, inpatient expense and total expense of SHI to estimate the project impacts (see Table 5). At baseline, the SHI reimbursements for outpatient expenditures of both groups were comparable (33.3 vs. 33.6, *P* > 0.05). The reimbursement ratios were 12.4 and 13.2%, respectively. The SHI reimbursed 642.2 (reimbursement ratio was 55.0%) yuan per capita for inpatient expenditure of the intervention group, which was significantly higher than the 384.5 (reimbursement ratio is 46.9%) of the control group. In total, the SHI of the intervention group reimbursed 258 yuan more than the control group (*P* < 0.05). At follow-up, the SHI of the intervention group reimbursed 125.3 yuan for per capita outpatient expenditure, which was significantly higher than the control group (*P* < 0.05). There was no statistical difference in inpatient expenditure reimbursement of both groups (*P* > 0.05). The per capita total expense of SHI of the intervention group was significantly more than the control group by 65.7 yuan (*P* < 0.05). The DID results showed that the SHI reimbursed 90.3 yuan more per capita for the outpatient expenditure. However, the per capita inpatient expenditure reimbursement and per capita total medical expenditure reimbursement decreased significantly by 282.6 (− 44.0%) yuan and 192.3 (− 28.5%) yuan, respectively (*P* < 0.05).

**Table 5:** DID estimation on the SHI per capita annual reimbursement expenditure

Outcome variable (y)	Baseline (1673 vs. 1673)			Follow-up (1673 vs. 1673)			
	Control $\left({\widehat{\upbeta}}_0\right)$	Intervention $\left({\widehat{\upbeta}}_0+\widehat{\gamma}\right)$	Diff (B)$\left(\widehat{\gamma}\right)$	Control $\left({\widehat{\upbeta}}_0+{\widehat{\upbeta}}_1\right)$	Intervention $\left({\widehat{\upbeta}}_0+{\widehat{\upbeta}}_1+\widehat{\gamma}+\widehat{\updelta}\right)$	Diff (FU) $\left(\widehat{\gamma}+\widehat{\updelta}\right)$	DIFF-IN-DIFF $\left(\widehat{\updelta}\right)$
Outpatient	33.3	33.6	0.3	34.7	125.3	90.6	90.3
*P*			0.951			0.031	0.018
Inpatient	384.5	642.2	257.7	475.5	450.6	−24.9	−282.6
*P*			0.029			0.830	0.016
Total	417.8	675.8	258.0	510.2	575.9	65.7	−192.3
*P*			0.005			0.048	0.010

### Patient per capita annual out-of-pocket expenditure

The per capita out-of-pocket outpatient, inpatient and total spending were estimated by DID method to represent changes in the economic burden of hypertension before and after the intervention. Table 6 illustrated the per capita out-of-pocket expenditures of the sampled patients. At baseline, there was no difference in outpatient and total out-of-pocket expenditures between both groups (235.9 vs. 221.8, 669.5 vs 747.2, respectively, *P* > 0.05). The inpatient out-of-pocket expenditure of the intervention group (525.4 yuan) was significantly more than the control group (433.6yuan, *P* < 0.05). At follow-up, both groups had comparable outpatient out-of-pocket expenditures (229.1 vs. 205.9, *P* > 0.05). The inpatient and total out-of-pocket expenditures of the intervention groups were more than the intervention group (435.4 vs 334.4, 664.5 vs 540.3, respectively, *P* < 0.5). The DID estimation showed that the outpatient out-of-pocket expenditure decreased 9.1 yuan per capita, but without statistical significance (*P* > 0.05). The intervention decreased the inpatient out-of-pocket expenditure and total out-of-pocket expenditure by 192.8 (− 36.7%) yuan per capita and 201.9 (− 29.9%) yuan per capita, respectively (*P* < 0.05).

**Table 6:** DID estimation on patient per capita annual out-of-pocket expenditure

Outcome variable (y)	Baseline (1673 vs. 1673)			Follow-up (1673 vs. 1673)			
	Control $\left({\widehat{\upbeta}}_0\right)$	Intervention $\left({\widehat{\upbeta}}_0+\widehat{\gamma}\right)$	Diff (BL) $\left(\widehat{\gamma}\right)$	Control $\left({\widehat{\upbeta}}_0+{\widehat{\upbeta}}_1\right)$	Intervention $\left({\widehat{\upbeta}}_0+{\widehat{\upbeta}}_1+\widehat{\gamma}+\widehat{\updelta}\right)$	Diff (FU) $\left(\widehat{\gamma}+\widehat{\updelta}\right)$	DIFF-IN-DIFF $\left(\widehat{\updelta}\right)$
Outpatient	235.9	221.8	−14.1	229.1	205.9	−23.2	−9.1
*P*			0.452			0.395	0.518
Inpatient	433.6	525.4	91.8	435.4	334.4	− 101	−192.8
*P*			0.049			0.043	0.027
Total	669.5	747.2	77.7	664.5	540.3	− 124.2	−201.9
*P*			0.085			0.041	0.022

### Health outcomes

Table 7 showed the impact of the intervention on the DBP and SBP of the patients. At baseline, both groups had comparable blood pressure (DBP 114.9 vs. 112.6, SBP 197.5 vs. 197.2, respectively, *P* > 0.05). At follow-up, the average DBP of the control group was 114.6 mmHg, which was significantly higher the 109.4 mmHg of the intervention group (*P* < 0.05). There was no significant difference in SBP between both groups (203.6 vs. 196.0, *P* > 0.05). The DID estimation results indicated that the intervention significantly decreased the DBP by 2.9 mmHg (*P* < 0.05) but had no significant impact on the SBP (− 7.9 mmHg, *P* > 0.05).

**Table 7:** DID estimation of Blood pressure (mmHg)

Outcome variable (y)	Baseline (1673 vs. 1673)			Follow-up (1673 vs. 1673)			
	Control $\left({\widehat{\upbeta}}_0\right)$	Intervention $\left({\widehat{\upbeta}}_0+\widehat{\gamma}\right)$	Diff (BL) $\left(\widehat{\gamma}\right)$	Control $\left({\widehat{\upbeta}}_0+{\widehat{\upbeta}}_1\right)$	Intervention $\left({\widehat{\upbeta}}_0+{\widehat{\upbeta}}_1+\widehat{\gamma}+\widehat{\updelta}\right)$	Diff (FU) $\left(\widehat{\gamma}+\widehat{\updelta}\right)$	DIFF-IN-DIFF $\left(\widehat{\updelta}\right)$
SBP	114.9	112.6	−2.3	114.6	109.4	−5.2	−2.9
*P*			0.855			0.043	0.011
DBP	197.5	197.2	0.3	203.6	196.0	−7.6	− 7.9
*P*			0.420			0.095	0.508

## Discussion

The results of our study indicated that outpatient services covered by SHI system called for a higher reimbursement ratio. Experience from countries where primary care stands at the center of medical care systems noted that primary care services could meet most health needs of the population [33, 34]. Primary care services were covered by medical insurance system to consolidate the primary care system [35]. However, due to the lack of health gatekeeper system (qualified GPs and family doctors) in rural China, primary care services were mainly, or if any, provided at village clinics, and outpatient department of township hospitals and county hospitals. Besides, outpatient facilities in rural China deserve a more comprehensive understanding. To our knowledge, although China has spent a lot to set up the Health Record System covering > 98% residents, the usage frequency of electronic health records is quite low [36, 37]. The main reason is that the information in health records is either seldom updated in time or filled in a mess. Health workers in villages and townships cannot give accurate and timely guidance on medical treatment according to the “dead records”. Therefore, outpatient caregivers provide not only patients basic medical treatment, but also health assessment and management services for many rural Chinese diseased population. In the current stage, most of the SHI policymakers apparently have not realized the fact that primary care services equate to outpatient services in rural China. The positive findings (− 201.9 yuan out-of-pocket expenditure, and − 2.9 mmHg in SBP) showed that SHI increasing the reimbursement ratio of outpatient services was beneficial to the performance of SHI. Therefore, outpatient services, rather than inpatient services, should be covered in priority by SHI in the future.

The intervention and DID estimation revealed that more use of outpatient services could help to control the rapid rising hospitalization. In this study, the pre-intervention hospitalization rates of the control group and the intervention group were 6.3 and 12.5%, respectively. The intervention decreased the hospitalization rate of the intervention group by 60.0%(0.075/0.125). One explanation for the significant reduction in hospitalization is the health needs of the sampled patients were met through the chronic disease management services provided by township physicians and outpatient treatments. Kim, Hyo-Jeong reported similar findings. Their analysis indicated that tertiary hospital outpatient coinsurance rate increase policy comparatively makes decrease effect on long-term healthcare utilization [38]. Another possible explanation is that unreasonable admissions among the intervention group were reduced. Across a range of healthcare settings, absurd admission was considered to be one of the leading contributors to the rapid rising hospitalization rate in rural China. For example, Chen Yingchun et al. found that the unreasonable admission rate in rural China was 12.57%, and the irrational admission rate in township hospitals and county hospital were 13.01 and 12.14%, respectively [39, 40]. In our study, the contracted physicians in the intervention group gave accurate suggestions on seeking medical treatment to the patient. Their unreasonable requests for admission might, therefore, be reduced.

In the intervention group, although the SHI fund budgeted 100 yuan per patient per year to the contracted physicians and their township hospitals, and 600 yuan per patient per year to cover the outpatient services, the inpatient expenditure, and total expenditure decreased significantly due to the 60% reduction of hospitalization rate. The decline of per capita total medical expenditures (− 27.7%), SHI expenditure (− 28.5%), patient out-of-pocket expenditure (− 29.9%), and the improved health outcomes (− 2.9 mmHg of DBP) in this study demonstrated that increasing the reimbursement ratio of outpatient expenditure contributed to the better performance of SHI. This positive finding provides crucial information for rural China and many other famous countries with scarce health resources.

Firstly, a universal coverage SHI with inadequate funds does not necessarily cover the inpatient services in priority for curing diseases. To the contrary, an emphasis on outpatient services is more effective and beneficial to improve the performance of SHI. This policy recommendation is also supported by numerous previous studies. For example, Mathew Mercuri, et al. found that, although it is not debatable that physicians can be an important source of variations (using no health services, outpatient care or hospitalization services) in health care services utilization [18, 41], physician-related factors are of lesser importance compared to other factors, explaining only 1% of the variance in hospital admission rates, 2% of the variance in overall resource use (2%), and 7% of the variance in overall laboratory costs [41]. The demand for outpatient care is quite responsive to economic factors, contrary to conventional beliefs about medical care [42].

Secondly, for many countries with SHI, hospitalization services are costing substantial funds and making health care costs continue to outpace inflation [43]. Therefore, the rapid rising hospitalization rate of the insured patients, especially those with chronic diseases, should be monitored strictly in the future reform. In fact, in the U.S. settings, with the passage of the Patient Protection and Affordable Care Act in 2010, there has been increased focus on hospitalization rate control. The federal government has a keen interest in unplanned readmissions of patients within 30 days from surgery. If a hospital’s readmission rate exceeded the Center for Medicare and Medicaid Services (CMS) parameters, then hospitals were penalized 1% of total revenues in 2013. This number rose to 2% in 2014 and 3% in 2015 [44].

Thirdly, although one of the main goals of China’s SHI is to protect the household from financial risk, the function of SHI should not be limited to compensate the medical expenditure. The present study indicated that SHI funds attributed to caregivers directly would motivate their initiative of providing primary care services. Meanwhile, the hospitalization was decreased dramatically. In this respect, SHI has the potential to reshape the delivery system and produce a value-based delivery system [45]. The main challenge for SHI policymakers is to adjust payment mode according to the target delivery system that their countries need.

The study’s strengths comprised the PSM method. The PSM balanced treatment and control samples on covariates of sex, age, incomes and health status without losing large numbers of observations (2083 to 1673 in the intervention group and 3152 to 1673 in the control group, see Table 2). Besides, we used the DID method to avoid the confounding effects caused by time [46] and estimate the net effects of the SHI performance-oriented reform. Our statistical analysis design was in accordance with Kim et al., and Quesnel-Vallée et al. In their studies, PSM method was used to get rid of selection bias by conditioning on confounding variables and past health status using a flexible semi-parametric specification. The PSM was then combined with a DID estimator that removes unobserved fixed effects via within-person comparisons over time as well as common period and ageing effects by comparing the trends of a treatment and control group [47, 48]. A limitation of our methodological approach was that PSM only accounts for observed (and observable) covariates (sex, age, incomes and health status). Factors that affecting assignment to treatment and outcome but that could not be observed were not accounted for in the matching procedure. Some hidden bias due to latent variables might remain after matching. Also, the SHI performance-oriented reform in Dangyang County only sampled patients with grade 3 hypertension. Although the substantial evidence in this study demonstrated that increasing outpatient services reimbursement ratio was associated with a better SHI performance, the impacts of these interventions on acute diseases were still debatable. Therefore, more SHI performance-oriented projects related to severe conditions should be designed and implemented across rural healthcare settings.

## Conclusion

In conclusion, to implement the ambitious strategy that China is now rolling out to improve its health system, SHI should emphasise the fundamental role of outpatient services for patients with chronic diseases. A higher reimbursement ratio for outpatient medical expenditures is a crucial support attracting patients to seek more outpatient services rather than “either no service or inpatient services”. China’s experience reveals that policymakers should be vigilant against the rapidly rising hospitalization wasting scarce medical resource and lowering the performance of SHI. The evidence of this article indicates that increasing the utilization of outpatient services contributed to reducing the hospitalization rate, total medical expenditures of patients and the SHI reimbursement expenditure. Moreover, better health outcomes of the patients associated with the more frequent medical services utilization would be obtained.

## Appendix

### PSM model setting

A propensity score is the probability of a unit (e.g. person, classroom, school) being assigned to a particular treatment given a set of observed covariates. Propensity scores are used to reduce selection bias by equating groups based on these covariates. For the marked individual I, suppose that we have a binary treatment T (*T* = 0 denotes being assigned to the control group, *T* = 1 denotes being attached to the control group), an outcome Y (Propensity Score), and background variables X (Sex, age, annual income and blood pressure). The propensity score is defined as the conditional probability of treatment given background variables:

1 $$ p(X)=\underline {\underline{def}}\Pr \left(\mathrm{T}=1\left|\mathrm{X}=\mathrm{x}\right.\right) $$

Let Y(0) and Y(1) denote the potential outcomes under control and treatment, respectively. Then treatment assignment is (conditionally) unconfounded if possible results are independent of treatment conditional on background variables X. This can be written compactly as:

2 $$ \mathrm{Y}(0),\mathrm{Y}(1)\perp \mathrm{T}\mid \mathrm{X} $$

Where ⊥ denotes statistical independence.

If unconfoundedness holds, then

3 $$ \mathrm{Y}(0),\mathrm{Y}(1)\perp \left(\mathrm{T}|\mathrm{p}\left(\mathrm{x}\right)\right) $$

### The definitions of the standardized difference

For dichotomous variables, the standardized difference is defined as

4 $$ d=\frac{100\left({p}_T-{p}_C\right)}{\sqrt{\left({p}_T\Big(1-{p}_T\right)+{p}_C\left(1-{p}_C\right)}} $$

In formula (4), p_T_ and p_C_ denote the proportion of treated and untreated subjects, respectively, for whom the condition denoted by the dichotomous variables is present.

For continuous variables, the standardized difference is defined as

5 $$ d=\frac{100\left({\overline{x}}_{treatment}-{\overline{x}}_{control}\right)}{\sqrt{\left({S}_{treatment}^2+{S}_{control}^2\right)/2}} $$

In formula (5), ${\overline{x}}_{treatment}$ and ${\overline{x}}_{control}$ denote the mean of the continuous variable in treated and untreated subjects, respectively. ${S}_{treatment}^2$ and ${S}_{control\kern0.5em }^2$denote the variances of the continuous variable in the treated and untreated subjects, respectively. It has been suggested that a standardized difference of greater than 10% represents meaningful imbalance in a given variable between treatment groups.
